# A Working Model of How Noroviruses Infect the Intestine

**DOI:** 10.1371/journal.ppat.1004626

**Published:** 2015-02-27

**Authors:** Stephanie M. Karst, Christiane E. Wobus

**Affiliations:** 1 Department of Molecular Genetics and Microbiology, Emerging Pathogens Institute, University of Florida, Gainesville, Florida, United States of America; 2 Department of Microbiology and Immunology, University of Michigan, Ann Arbor, Michigan, United States of America; The Fox Chase Cancer Center, UNITED STATES

## Introduction

Human noroviruses (HuNoVs) cause a majority of gastroenteritis outbreaks across the globe and are the leading cause of severe childhood diarrhea and foodborne disease outbreaks in the United States [1,2]. In impoverished countries, they are estimated to cause over one million clinic visits and 200,000 deaths in young children annually [3]. However, the mechanisms used by noroviruses (NoVs) to infect the intestinal tract and cause disease are not well understood, primarily due to the paucity of cell culture and animal model systems. Recent major advances in developing such models now leave the field poised to tackle these critical questions. The goal of this opinion article is to propose a working model of early steps involved in intestinal infection by NoVs. In this model, NoVs bind carbohydrates on the surface of specific members of the intestinal microbiota and/or enterocytes and are then transcytosed across the intestinal epithelial barrier to gain access to their target immune cells. Evidence supporting each step of this model will be discussed. We also include a brief discussion of how NoVs cause disease as it relates to our model.

### NoVs Are Transcytosed Across Enterocytes in the Absence of Viral Replication

**Fig 1:**
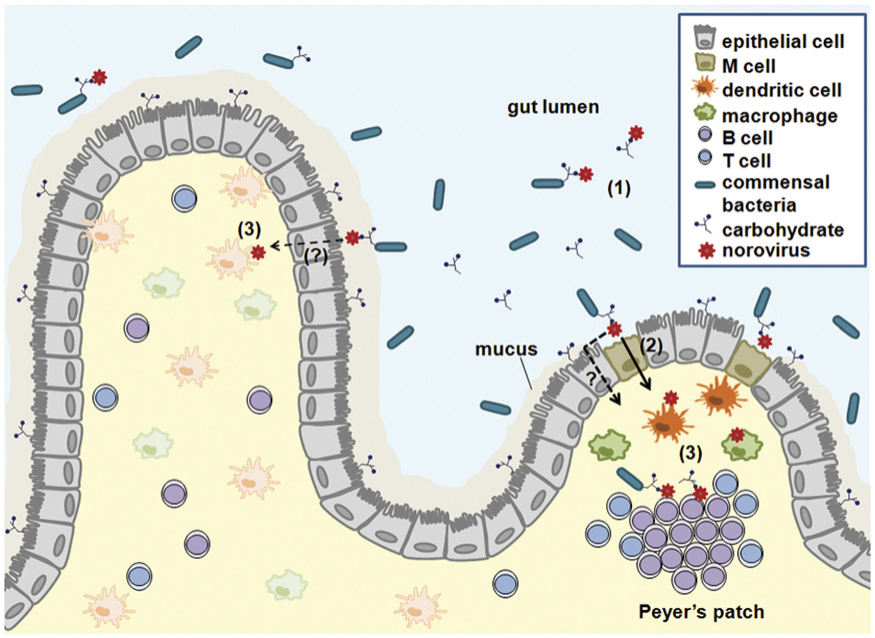
A working model for NoV intestinal infection. Multiple studies demonstrate that NoVs bind carbohydrates. These carbohydrates are expressed on enterocytes and secreted into the gut lumen. Furthermore, enteric bacteria can express similar carbohydrates. NoVs may bind to such carbohydrates in any of these contexts **(1)**. NoVs are then transcytosed across the intestinal epithelium via M cells **(2)** and additional as-yet-to-be-identified pathways. Following transcytosis, NoVs infect dendritic cells, macrophages, and B cells **(3)**. Depending on the species, infection can occur in the presence or absence of carbohydrates. Free carbohydrates or bacterially expressed carbohydrates may be cotranscytosed with the virus. Immune cell infection and putative concomitant viral-bacterial antigen presentation during NoV infections could have significant consequences on the nature and magnitude of antiviral immune responses.

HuNoV and murine NoVs (MuNoV) are transcytosed across intestinal epithelial cells in vitro [4,5], although they have not been shown to productively infect these cells in immunocompetent hosts (reviewed in [6]). Transcytosis of MuNoV across polarized murine intestinal epithelial cell monolayers does not disrupt tight junctions, is enhanced by B cell coculture, and is mediated by cells with characteristics of microfold (M) cells [4], a specialized cell type within the intestine responsible for sampling particulate antigen [7]. In a similar system, HuNoV virus-like particles were visualized on the basolateral side of cell nuclei from polarized Caco-2 cells [5], suggesting transport of particles through epithelial cells. However, whether particles were released from cells, whether particle transport modulated tight junction integrity, or whether a specialized cell type such as M cells mediated this process was not investigated. The importance of M cells for the efficient initiation of MuNoV infection in vivo was subsequently demonstrated by infecting mice depleted of M cells and observing reductions in viral titers in the intestine [8]. Furthermore, this partial, in contrast to complete, reduction of MuNoV infection in M cell-depleted mice suggests the presence of additional viral uptake routes across the intestinal barrier. Reovirus, a double-stranded RNA virus that infects enterocytes, similarly requires M cells for efficient infection [8], and other enteric pathogens also exploit M cells to infect the host [9]. Hence, we speculate that similar mechanisms are used by HuNoV to cross the intestinal epithelial barrier (Fig 1).

### NoVs Infect Innate Immune Cells

Upon crossing the epithelial barrier, viral particles next encounter immune cells in the lamina propria and lymphoid follicles, including Peyer’s patches (Fig 1). The evidence that NoVs infect immune cells is numerous, although intestinal epithelial cells are also infected in case of bovine NoV [10]. MuNoV lytically replicates in antigen-presenting dendritic cells and macrophages in vitro [11]. In vivo, MuNoV antigen is detectable in cells morphologically resembling dendritic cells and macrophages and in cells positive for the macrophage marker F4/80 [11,12]. Although one report failed to observe HuNoV replication in peripheral blood-derived macrophages and dendritic cells in vitro [13], HuNoV appears to target intestinal immune cells in vivo consistent with the tropism of MuNoV: viral antigen was detected in intestinal lamina propria cells from a biopsy sample of a HuNoV-infected person [13]; and inactivated HuNoV particles bind to lamina propria cells in human intestinal tissue sections [14]. Additional support comes from animal models of HuNoV infection: chimpanzees and immunodeficient mice infected with a HuNoV contain viral antigens in cells resembling, or confirmed to be, dendritic cells or macrophages [15,16]. Finally, monkeys infected with genetically related recoviruses and bovine NoV also contain virus antigen-positive intestinal lamina propria cells [10,17]. While detection of viral intermediates in antigen-presenting cells in vivo may be due to phagocytosis of apoptotic epithelial cells infected by bovine NoV, viral antigen was not detected in enterocytes in any of the other examples. This, along with the ability of MuNoV to infect innate immune cells in vitro, supports that these cell types are bona fide NoV targets. It is likely that infection of antigen-presenting cells influences the immune outcome to infection, which was recently demonstrated for MuNoV [18].

### NoVs Infect Adaptive Immune Cells

In addition to macrophages and dendritic cells, B cells were recently identified as targets of NoV infection [19]. Both HuNoV and MuNoV productively infect B cell lines in vitro, establishing the first cell culture system for HuNoV. B cell infection appears to be distinct from macrophage or dendritic cell infection in that no cytopathic effect is observed in infected cultures and is distinct from lytic infection of intestinal B cells by rotavirus [20]. Whether this is due to true noncytopathic B cell infection (precedence exists for noncytopathic infection by a nonenveloped, positive-sense RNA virus [21]) or the low infectivity of this cell type remains to be determined. B cells are also target cells in vivo: MuNoV titers are reduced in mice lacking B cells, and MuNoV antigen is detected in B cell zones of Peyer’s patches of infected interferon- and interleukin-10-deficient mice [22,23]. MuNoV genome and nonstructural protein are detected in Peyer’s patch B cells of infected mice [19]; and HuNoV-infected chimpanzees contain virus antigen-positive B cells in the small intestine [16]. Given that several MuNoV strains can persistently infect mature B cells in vitro [19], we speculate that B cells may also provide a reservoir for persistent MuNoV infections in vivo.

### Enteric Bacteria Serve as a Co-Factor for NoV Infection

Enteric bacteria can enhance viral infections [24] since poliovirus, reovirus, and mouse mammary tumor virus infections are reduced in antibiotic-treated or germ-free mice [25,26]. Similarly, antibiotic treatment of mice resulted in a significant reduction in MuNoV yield in the intestine when compared to untreated mice [19]. Thus, commensal bacteria can stimulate NoV infections in vivo and may influence the immune response to viral infection. Although enteric bacteria are not required for MuNoV infection in vitro [11], they significantly enhance HuNoV infection of B cells in vitro [19].

The mechanism(s) of bacterial enhancement of enteric virus infection is not well understood. While binding to bacterial lipopolysaccharide (LPS) is one mechanism [26,27], LPS does not enhance HuNoV infection of B cells in vitro [19]. Instead, histo-blood group antigen (HBGA)-expressing bacteria and free HBGA stimulate HuNoV infection of B cells, while non-HBGA-expressing bacteria do not [19]. HBGAs are neutral carbohydrates found on proteins or lipids that are bound by individual HuNoV strains and their expression correlates with a person’s susceptibility to infection (reviewed e.g., in [28]). Virus binding to HBGAs expressed on host enterocytes has been thought to facilitate retention in the intestine and to counter the movement of particles via peristalsis. However, expression of appropriate HBGAs on enterocytes in culture does not mediate infection [29]. Interestingly, certain pathogenic and commensal enteric bacteria also express carbohydrates indistinguishable from human HBGAs [30–34], and HuNoV particles bind to HBGA-expressing bacteria [35]. Interaction of HuNoV with free or bacteria-bound HBGAs enhances attachment to, and infection of, B cells [19]. While an interaction between specific bacteria and MuNoV has not been shown to date, MuNoV binds carbohydrates such as sialic acids [36] which are abundant on the surface of enteric bacteria [37]. Thus, we speculate that NoVs bind specific carbohydrates on the surface of certain bacteria instead of, or in addition to, enterocytes to enhance infection of the host (Fig 1).

Bacteria may also play additional roles in vivo by enhancing the transcytosis of NoVs across the intestinal epithelium. While HuNoV and MuNoV can be transcytosed across polarized cells in the absence of bacteria in vitro [4,5], there are additional physical barriers (e.g., a thick mucus lining) impeding their access to the epithelium in the complex environment of the intestinal lumen. To overcome such physical barriers, we hypothesize that NoVs may bind to motile bacteria that can traverse the mucus layer [38]. In addition, the host continuously samples its luminal cargo; for example, commensal bacteria in complex with secretory immunoglobulin A (sIgA) are taken up via Peyer’s patch-associated M cells and delivered to underlying dendritic cells and macrophages [39]. Viral particles bound to bacteria could thus be delivered to permissive immune cells. Since sIgA complexes are generally anti-inflammatory [40], this might account for the mild inflammation observed during MuNoV infection [23]. Conversely, it is possible that NoVs actively drive transcytosis of commensal bacteria. Studies that provide mechanistic insights into the bacterial enhancement of NoV infection are clearly needed and promise to provide important insights into the interplay between the intestinal microbiota, enteric viruses, and the host.

### Gaps in Understanding NoV Pathogenesis

In this section, we briefly speculate how the proposed model might relate to unanswered questions in NoV pathogenesis. Given the length restrictions, we limit our discussion to i) viral shedding and ii) mechanisms of gastroenteritis.

First, the source of virus shed in the feces is one conundrum that exists in NoV pathogenesis. Shedding varies greatly with peak titers ranging between 10^5^–10^9^ genome copies/g of feces and lasting days to months [41], and high viral titers likely contribute to the explosive nature of NoV outbreaks. However, this high-level shedding appears inconsistent with the low-level viral replication in cultured B cells [19]. A likely explanation for this discrepancy is that B cell lines do not entirely mirror the properties of intestinal B cells in vivo. Additionally, intestinal macrophages and dendritic cells could support high levels of NoV replication similar to MuNoV infection [11]. Finally, it should be noted that only sections of small intestines have been analyzed for HuNoV antigens thus far. It is possible that a population of highly permissive cells in other regions of the intestine (e.g., cecum or colon) or an unrecognized virus reservoir at an extraintestinal site (e.g., liver with shedding into bile fluids) is responsible for the robust viral shedding.

Second, one question raised by our model is how NoVs cause gastroenteritis in the absence of enterocyte infection. We propose that one or more of the following mechanisms may be at play: (1) Infection of immune cells could trigger the release of pathologic levels of proinflammatory cytokines, although NoV infections are only modestly inflammatory based on available data [6]. (2) NoVs could encode a viral enterotoxin similar to the rotavirus NSP4 protein [42], and secretion of this enterotoxin from infected immune cells could act on enterocytes basally to cause epithelium dysfunction. (3) NoVs could stimulate the transcytosis of commensal bacteria that are generally considered nonpathogenic because they cannot breach the intestinal epithelium. In this scenario, pathologic mechanisms encoded by the bacteria would contribute to NoV-associated disease. (4) NoV infection of intestinal macrophages could cause functional changes that result in altered motor function, since intestinal macrophages closely interact with the enteric nervous system and regulate gut motility [43–45]. This crosstalk between macrophages and neurons is also regulated by the microbiota [43]. To explore these scenarios, studies in NoV animal models displaying disease symptoms and, particularly in case of the latter two, including a functional microbiota, are needed in the future.

## Concluding Remarks

The NoV field has made great strides in recent years elucidating the cell tropism and mechanisms of intestinal infection, although these areas remain incompletely defined and sometimes controversial. Herein, we have evaluated and integrated available data often from in vitro and mouse studies to propose a working model of intestinal infection for NoVs: in this model, we propose that NoVs bind to bacterial and/or host carbohydrates within the gut lumen, transcytose across the intestinal epithelium, and are delivered to target immune cells in the lamina propria. We further speculate that cotranscytosed carbohydrate (free or as part of bacteria) can directly stimulate NoV attachment to, and infection of, these target cells. Future studies are needed to determine whether NoV infections in all host species (e.g., humans, mice, dogs, and pigs) follow a similar pattern such as the one proposed herein or vary significantly in their infection route(s).

Collectively, it is our hope that by sharing this model we will facilitate experimental approaches to test individual aspects of the model and that such studies will advance our understanding of NoVs and enteric viruses in general.

## References

[1] Payne DC, Vinje J, Szilagyi PG, Edwards KM, Staat MA, et al (2013) Norovirus and medically attended gastroenteritis in U.S. children. N Engl J Med 368: 1121–1130. 10.1056/NEJMsa1206589 23514289 PMC4618551

[2] Green KY (2013) Caliciviridae: The Noroviruses In: Knipe DM, Howley P.M., Cohen J.I., Griffin D.I., Lamb R.A., Martin M.A., Racaniello V.R., and Roizman B., editor. Fields Virology. Philadelphia: Lippincott Williams & Wilkins, a Wolters Kluwer Business pp. 582–608.

[3] Patel MM, Widdowson MA, Glass RI, Akazawa K, Vinje J, et al (2008) Systematic literature review of role of noroviruses in sporadic gastroenteritis. Emerg Infect Dis 14: 1224–1231. 10.3201/eid1408.071114 18680645 PMC2600393

[4] Gonzalez-Hernandez MB, Liu T, Blanco LP, Auble H, Payne HC, et al (2013) Murine norovirus transcytosis across an in vitro polarized murine intestinal epithelial monolayer is mediated by M-like cells. J Virol 87: 12685–12693. 10.1128/JVI.02378-13 24049163 PMC3838167

[5] Marionneau S, Ruvoen N, Le Moullac-Vaidye B, Clement M, Cailleau-Thomas A, et al (2002) Norwalk virus binds to histo-blood group antigens present on gastroduodenal epithelial cells of secretor individuals. Gastroenterology 122: 1967–1977. 12055602 10.1053/gast.2002.33661 PMC7172544

[6] Karst SM, Wobus CE, Goodfellow IG, Green KY, Virgin HW (2014) Advances in norovirus biology. Cell Host Microbe 15: 668–680. 10.1016/j.chom.2014.05.015 24922570 PMC4113907

[7] Mabbott NA, Donaldson DS, Ohno H, Williams IR, Mahajan A (2013) Microfold (M) cells: important immunosurveillance posts in the intestinal epithelium. Mucosal Immunol 6: 666–677. 10.1038/mi.2013.30 23695511 PMC3686595

[8] Gonzalez-Hernandez MB, Liu T, Payne HC, Stencel-Baerenwald JE, Ikizler M, et al (2014) Efficient Norovirus and Reovirus Replication in the Mouse Intestine Requires Microfold (M) Cells. J Virol 88: 6934–6943. 10.1128/JVI.00204-14 24696493 PMC4054386

[9] Miller H, Zhang J, Kuolee R, Patel GB, Chen W (2007) Intestinal M cells: the fallible sentinels? World J Gastroenterol 13: 1477–1486. 17461437 10.3748/wjg.v13.i10.1477 PMC1876659

[10] Otto PH, Clarke IN, Lambden PR, Salim O, Reetz J, et al (2011) Infection of calves with bovine norovirus GIII.1 strain Jena virus: an experimental model to study the pathogenesis of norovirus infection. J Virol 85: 12013–12021. 10.1128/JVI.05342-11 21880760 PMC3209315

[11] Wobus CE, Karst SM, Thackray LB, Chang KO, Sosnovtsev SV, et al (2004) Replication of Norovirus in cell culture reveals a tropism for dendritic cells and macrophages. PLoS Biol 2: e432 15562321 10.1371/journal.pbio.0020432 PMC532393

[12] Ward JM, Wobus CE, Thackray LB, Erexson CR, Faucette LJ, et al (2006) Pathology of immunodeficient mice with naturally occurring murine norovirus infection. Toxicol Pathol 34: 708–715. 17074739 10.1080/01926230600918876

[13] Lay MK, Atmar RL, Guix S, Bharadwaj U, He H, et al (2010) Norwalk virus does not replicate in human macrophages or dendritic cells derived from the peripheral blood of susceptible humans. Virology 406: 1–11. 10.1016/j.virol.2010.07.001 20667573 PMC2933743

[14] Chan MC, Ho WS, Sung JJ (2011) In vitro whole-virus binding of a norovirus genogroup II genotype 4 strain to cells of the lamina propria and Brunner’s glands in the human duodenum. J Virol 85: 8427–8430. 10.1128/JVI.05016-11 21680503 PMC3147981

[15] Taube S, Kolawole AO, Hohne M, Wilkinson JE, Handley SA, et al (2013) A mouse model for human norovirus. MBio 4: e00450–13. 10.1128/mBio.00450-13 23860770 PMC3735125

[16] Bok K, Parra GI, Mitra T, Abente E, Shaver CK, et al (2011) Chimpanzees as an animal model for human norovirus infection and vaccine development. Proc Natl Acad Sci U S A 108: 325–330. 10.1073/pnas.1014577107 21173246 PMC3017165

[17] Sestak K, Feely S, Fey B, Dufour J, Hargitt E, et al (2012) Experimental inoculation of juvenile rhesus macaques with primate enteric caliciviruses. PLoS ONE 7: e37973 10.1371/journal.pone.0037973 22666426 PMC3364207

[18] Zhu S, Regev D, Watanabe M, Hickman D, Moussatche N, et al (2013) Identification of immune and viral correlates of norovirus protective immunity through comparative study of intra-cluster norovirus strains. PLoS Pathog 9: e1003592 10.1371/journal.ppat.1003592 24039576 PMC3764223

[19] Jones MK, Watanabe M, Zhu S, Graves CL, Keyes LR, et al (2014) Enteric bacteria promote human and mouse norovirus infection of B cells. Science 346: 755–759. 10.1126/science.1257147 25378626 PMC4401463

[20] Narvaez CF, Franco MA, Angel J, Morton JM, Greenberg HB (2010) Rotavirus differentially infects and polyclonally stimulates human B cells depending on their differentiation state and tissue of origin. J Virol 84: 4543–4555. 10.1128/JVI.02550-09 20164228 PMC2863723

[21] Feng Z, Hensley L, McKnight KL, Hu F, Madden V, et al (2013) A pathogenic picornavirus acquires an envelope by hijacking cellular membranes. Nature 496: 367–371. 10.1038/nature12029 23542590 PMC3631468

[22] Basic M, Keubler LM, Buettner M, Achard M, Breves G, et al (2014) Norovirus triggered microbiota-driven mucosal inflammation in interleukin 10-deficient mice. Inflamm Bowel Dis 20: 431–443. 10.1097/01.MIB.0000441346.86827.ed 24487272

[23] Mumphrey SM, Changotra H, Moore TN, Heimann-Nichols ER, Wobus CE, et al (2007) Murine norovirus 1 infection is associated with histopathological changes in immunocompetent hosts, but clinical disease is prevented by STAT1-dependent interferon responses. J Virol 81: 3251–3263. 17229692 10.1128/JVI.02096-06 PMC1866040

[24] Wilks J, Golovkina T (2012) Influence of microbiota on viral infections. PLoS Pathog 8: e1002681 10.1371/journal.ppat.1002681 22615558 PMC3355081

[25] Kuss SK, Best GT, Etheredge CA, Pruijssers AJ, Frierson JM, et al (2011) Intestinal microbiota promote enteric virus replication and systemic pathogenesis. Science 334: 249–252. 10.1126/science.1211057 21998395 PMC3222156

[26] Kane M, Case LK, Kopaskie K, Kozlova A, MacDearmid C, et al (2011) Successful transmission of a retrovirus depends on the commensal microbiota. Science 334: 245–249. 10.1126/science.1210718 21998394 PMC3519937

[27] Robinson CM, Jesudhasan PR, Pfeiffer JK (2014) Bacterial lipopolysaccharide binding enhances virion stability and promotes environmental fitness of an enteric virus. Cell Host Microbe 15: 36–46. 10.1016/j.chom.2013.12.004 24439896 PMC3920179

[28] Tan M, Jiang X (2011) Norovirus-host interaction: multi-selections by human histo-blood group antigens. Trends Microbiol 19: 382–388. 10.1016/j.tim.2011.05.007 21705222 PMC3149758

[29] Guix S, Asanaka M, Katayama K, Crawford SE, Neill FH, et al (2007) Norwalk virus RNA is infectious in mammalian cells. J Virol 81: 12238–12248. 17855551 10.1128/JVI.01489-07 PMC2168986

[30] Andersson M, Carlin N, Leontein K, Lindquist U, Slettengren K (1989) Structural studies of the O-antigenic polysaccharide of Escherichia coli O86, which possesses blood-group B activity. Carbohydr Res 185: 211–223. 2471591 10.1016/0008-6215(89)80036-9

[31] Aspinall GO, Monteiro MA (1996) Lipopolysaccharides of Helicobacter pylori strains P466 and MO19: structures of the O antigen and core oligosaccharide regions. Biochemistry 35: 2498–2504. 8652594 10.1021/bi951853k

[32] Rasko DA, Wang G, Monteiro MA, Palcic MM, Taylor DE (2000) Synthesis of mono- and di-fucosylated type I Lewis blood group antigens by Helicobacter pylori. Eur J Biochem 267: 6059–6066. 10998067 10.1046/j.1432-1327.2000.01683.x

[33] Springer GF, Williamson P, Brandes WC (1961) Blood Group Activity of Gram-Negative Bacteria. J Exp Med 113: 1077–1093. 19867191 10.1084/jem.113.6.1077 PMC2137423

[34] Yi W, Shao J, Zhu L, Li M, Singh M, et al (2005) Escherichia coli O86 O-antigen biosynthetic gene cluster and stepwise enzymatic synthesis of human blood group B antigen tetrasaccharide. J Am Chem Soc 127: 2040–2041. 15713070 10.1021/ja045021y

[35] Miura T, Sano D, Suenaga A, Yoshimura T, Fuzawa M, et al (2013) Histo-blood group antigen-like substances of human enteric bacteria as specific adsorbents for human noroviruses. J Virol 87: 9441–9451. 10.1128/JVI.01060-13 23804639 PMC3754087

[36] Taube S, Perry JW, Yetming K, Patel SP, Auble H, et al (2009) Ganglioside-linked terminal sialic acid moieties on murine macrophages function as attachment receptors for murine noroviruses. J Virol 83: 4092–4101. 10.1128/JVI.02245-08 19244326 PMC2668497

[37] Vimr ER, Kalivoda KA, Deszo EL, Steenbergen SM (2004) Diversity of microbial sialic acid metabolism. Microbiol Mol Biol Rev 68: 132–153. 15007099 10.1128/MMBR.68.1.132-153.2004 PMC362108

[38] Bansil R, Celli JP, Hardcastle JM, Turner BS (2013) The Influence of Mucus Microstructure and Rheology in Helicobacter pylori Infection. Front Immunol 4: 310 10.3389/fimmu.2013.00310 24133493 PMC3794295

[39] Rol N, Favre L, Benyacoub J, Corthesy B (2012) The role of secretory immunoglobulin A in the natural sensing of commensal bacteria by mouse Peyer’s patch dendritic cells. J Biol Chem 287: 40074–40082. 10.1074/jbc.M112.405001 23027876 PMC3501041

[40] Mkaddem SB, Christou I, Rossato E, Berthelot L, Lehuen A, et al (2014) IgA, IgA receptors, and their anti-inflammatory properties. Curr Top Microbiol Immunol 382: 221–235. 10.1007/978-3-319-07911-0_10 25116102

[41] Teunis PF, Sukhrie FH, Vennema H, Bogerman J, Beersma MF, et al (2014) Shedding of norovirus in symptomatic and asymptomatic infections. Epidemiol Infect: 1–8. 25336060 10.1017/S095026881400274X PMC9507237

[42] Ball JM, Mitchell DM, Gibbons TF, Parr RD (2005) Rotavirus NSP4: a multifunctional viral enterotoxin. Viral Immunol 18: 27–40. 15802952 10.1089/vim.2005.18.27

[43] Muller PA, Koscso B, Rajani GM, Stevanovic K, Berres ML, et al (2014) Crosstalk between muscularis macrophages and enteric neurons regulates gastrointestinal motility. Cell 158: 300–313. 10.1016/j.cell.2014.04.050 25036630 PMC4149228

[44] Wouters MM, Boeckxstaens GE (2011) Neuroimmune mechanisms in functional bowel disorders. Neth J Med 69: 55–61. 21411840

[45] Wehner S, Behrendt FF, Lyutenski BN, Lysson M, Bauer AJ, et al (2007) Inhibition of macrophage function prevents intestinal inflammation and postoperative ileus in rodents. Gut 56: 176–185. 16809419 10.1136/gut.2005.089615 PMC1856749

[46] Kernbauer E, Ding Y, Cadwell K (2014) An enteric virus can replace the beneficial function of commensal bacteria. Nature 516: 94–98. 10.1038/nature13960 25409145 PMC4257755

[47] Baldridge MT, Nice TJ, McCune BT, Yokoyama CC, Kambal A, et al (2014) Commensal microbes and interferon-lambda determine persistence of enteric murine norovirus infection. Science 347: 266–269. 10.1126/science.1258025 25431490 PMC4409937

